# Sharing Physician Notes Through an Electronic Portal is Associated With Improved Medication Adherence: Quasi-Experimental Study

**DOI:** 10.2196/jmir.4872

**Published:** 2015-10-08

**Authors:** Eric Wright, Jonathan Darer, Xiaoqin Tang, Jason Thompson, Lorraine Tusing, Alan Fossa, Tom Delbanco, Long Ngo, Jan Walker

**Affiliations:** ^1^ Geisinger Health System Center for Health Research Danville, PA United States; ^2^ Wilkes University Department of Pharmacy Practice Wilkes-Barre, PA United States; ^3^ Geisinger Health System Institute for Advanced Applications Danville, PA United States; ^4^ Beth Israel Deaconess Medical Center Division of General Medicine and Primary Care Harvard Medical School Boston, MA United States

**Keywords:** patient portal, adherence, progress notes, hypertension, hyperlipidemia

## Abstract

**Background:**

In surveys, interviews, and focus groups, patients taking medications and offered Web portal access to their primary care physicians’ (PCPs) notes report improved adherence to their regimens. However, objective confirmation has yet to be reported.

**Objective:**

To evaluate the association between patient Internet portal access to primary care physician visit notes and medication adherence.

**Methods:**

This study is a retrospective comparative analysis at one site of the OpenNotes quasi-experimental trial. The setting includes primary care practices at the Geisinger Health System (GHS) in Danville, Pennsylvania. Participants include patients 18 years of age or older with electronic portal access, GHS primary care physicians, and Geisinger health plan insurance, and taking at least one antihypertensive or antihyperlipidemic agent from March 2009 to June 2011. Starting in March 2010, intervention patients were invited and reminded to read their PCPs' notes. Control patients also had Web portal access throughout, but their PCPs' notes were not available. From prescription claims, adherence was assessed by using the proportion of days covered (PDC). Patients with a PDC ≥.80 were considered adherent and were compared across groups using generalized linear models.

**Results:**

A total of 2147 patients (756 intervention participants, 35.21%; 1391 controls, 64.79%) were included in the analysis. Compared to those without access, patients invited to review notes were more adherent to antihypertensive medications—adherence rate 79.7% for intervention versus 75.3% for control group; adjusted risk ratio, 1.06 (95% CI 1.00-1.12). Adherence was similar among patient groups taking antihyperlipidemic agents—adherence rate 77.6% for intervention versus 77.3% for control group; adjusted risk ratio, 1.01 (95% CI 0.95-1.07).

**Conclusions:**

Availability of notes following PCP visits was associated with improved adherence by patients prescribed antihypertensive, but not antihyperlipidemic, medications. As the use of fully transparent records spreads, patients invited to read their clinicians’ notes may modify their behaviors in clinically valuable ways.

## Introduction

Advances in health information technology, including secure patient portals associated with electronic health records (EHRs), create new opportunities for providers, patients, and families to interact and share information. Moreover, patient reports suggest strongly that unprecedented access to clinical information, including the notes clinicians write, may improve adherence to medical regimens [1].

With the goal of promoting engagement and communication between patients and clinicians, primary care physicians (PCPs) participating in the OpenNotes initiative invited their patients to read progress notes through a secure patient portal. A large majority of patients in each site chose to read their notes and reported feeling more in control of their health care, being more prepared for visits, and understanding their medical conditions better [2]. Of particular interest, they reported improving their adherence to medications. More than two-thirds of patients taking medications who responded to postintervention surveys reported that access to their PCPs' notes helped them “take their medications better” [3]. A similar small pilot study in heart failure patients supports a correlation between medical record access and improved adherence, but beyond self-report, no large studies have examined whether adherence does indeed change [4].

Nonadherence to medications is a major and potentially modifiable risk factor for heavy utilization of health care resources, disease progression, and mortality [5]. For newly prescribed medications, 10 to 30% of patients never fill the original prescription, and for some medications prescribed to manage chronic illness, nonadherence rates can approach 50% at 6 months [6]. Low adherence to medications can affect both patient outcomes and health care costs, with some estimating US $100 billion in excess US health care system costs per year [7]. Improving adherence has long been a challenge for both clinicians and patients; although some interventions targeting the patient, provider, and the health delivery system have demonstrated higher adherence, they are often limited to defined diseases or patient populations, are frequently costly, and the results from different studies vary considerably [8,9]. Ideally, interventions designed to encourage adherence would take into account patient and provider preferences, would be inexpensive, would both work across and be tailored to multiple health conditions, and would be replicable across settings.

The Geisinger Health System (GHS), one of three sites in the OpenNotes inquiry, offers health insurance to about a third of the patients it manages, and among them we had the opportunity to examine to what degree patients filled and refilled prescriptions written by their PCPs. Two conditions with high frequency in this population are hypertension and hyperlipidemia. With medications prescribed for these conditions as markers, the goal of our study was to evaluate whether a reminder to read, and the ready availability of, PCP progress notes did indeed affect patient medication adherence.

## Methods

### Design Overview

The OpenNotes study was a multicenter, prospective quasi-experimental study of participants invited to read PCP notes through electronic portals following their visits [2]. In 2010, the intervention was initiated at three sites: Beth Israel Deaconess Medical Center in Boston, Harborview Medical Center in Seattle, and the GHS in Pennsylvania. Surveys completed by patients and physicians, both those volunteering to participate in the intervention and those declining participation, were previously reported [1,3]. For this inquiry, we conducted a quasi-experimental comparative study among patients within the GHS. The study was conducted with Institutional Review Board (IRB) review at GHS; it was determined to be exempt due to the use of deidentified data.

### Setting and Participants

GHS is a fully integrated health care system that provides health services to more than 2.6 million people in a 44-county region in central and northeast Pennsylvania. In GHS’s area of coverage, 29 counties are designated as rural, with 18% of the population over 65 years of age. An EHR system has been fully operational within GHS practices since 2001. Information from the EHR along with financial, health plan prescription and medical claims, patient satisfaction, and high-use third-party reference datasets are backed up on a comprehensive enterprise data warehouse every 24 hours.

Patients from GHS included those cared for by 24 PCPs in 14 clinic locations who volunteered to participate in the OpenNotes evaluation, and those cared for by 78 PCPs in the same clinics who declined to participate. There was at least one nonparticipating PCP in each clinic where a participating PCP practiced. Patients were included if they were 18 to 89 years of age, assigned a GHS PCP, had an active portal account (called MyGeisinger), and had Geisinger health plan insurance 1 year prior to the intervention (March 1, 2009-February 28, 2010 or July 1, 2009-June 30, 2010) and throughout the time period of the intervention (March 1, 2010-February 28, 2011 or July 1, 2010-June 30, 2011). Patients were included if they had a prescription claim filed for an antihypertensive or antihyperlipidemic medication during the year prior to deploying OpenNotes (baseline), and during the study year (follow-up) of the analysis.

### Intervention

Intervention patients were offered access to their PCP notes via the MyGeisinger Web portal. Following signature of a note by a PCP documenting an encounter, patients received an email message sent to their personal email address notifying them of a portal message. The message included a direct link to the note section of the portal. In addition, prior to their next scheduled PCP appointment, a MyGeisinger reminder message was sent, encouraging the patient to review the previous PCP note(s) prior to the appointment. Control patients were those meeting the inclusion criteria above and were listed as a patient from the panel of nonparticipating physicians within a practice where at least one OpenNotes physician was participating. Control patients were also MyGeisinger users, but did not have access to their PCP notes. All other portal functionality, including access to lab results, medication and problem lists, appointment information, and correspondence with providers, was available to both intervention and control patients throughout the study period. Because there were more PCPs who did not volunteer to participate, we expected to have more control than intervention patients.

### Outcomes and Follow-Up

We compared adherence to two classes of medications, antihypertensive and antihyperlipidemic agents, both commonly prescribed in our primary care population and for which previous interventional studies have evaluated adherence through prescription records [10-13]. From prescription claims, adherence was assessed by using the proportion of days covered (PDC) [14]. The PDC was defined as the number of days covered, based on the medication fill date and days of supply, multiplied by the number of claims in the defined period, divided by the length of the study period (365 days). Drugs within one subclass were included in the same PDC calculation. If more than one subclass claim was identified per patient during the period of interest (eg, atenolol and hydrochlorothiazide), separate PDCs were calculated for each and then averaged together to arrive at one antihypertensive or antihyperlipidemic PDC per patient per period of interest. The value of a PDC is bounded between 0 and 1 and is often converted to percentages between 0 and 100%. Our primary outcome was adherence during the follow-up year. We used a breakpoint of ≥80% PDC for adherence, a percentage widely accepted in the literature as appropriate for designating adherent versus nonadherent patients for these classes of medications [15,16]. We evaluated rates of adherence versus nonadherence in the two time periods (baseline and follow-up).

### Statistical Analysis

We calculated means and standard deviations for symmetric continuous variables, medians and interquartile ranges (IQRs) for asymmetric continuous variables, and frequencies and percentages for categorical variables. Baseline comparisons between intervention and control groups were made using a two-sample *t* test or Kruskal-Wallis test, and Pearson's chi-square tests. We used a dichotomous variable of *adherent* as the outcome variable, with a value of 1 assigned to patients having ≥80% PDC. Multivariable logistic regression was used to detect differences between groups, controlling for potential confounders which included diabetes, body mass index (BMI), and the number of primary care visits per year.

In a secondary analysis to determine whether patients changed adherence status pre- and postintervention, a four-level outcome variable was created: nonadherent to adherent, adherent to adherent, nonadherent to nonadherent, and adherent to nonadherent. A multinomial logistic regression model was used to test for differences between groups. All statistical analyses were performed using SAS software, version 9.3 (SAS Institute, Cary, North Carolina).

## Results

**Figure 1:**
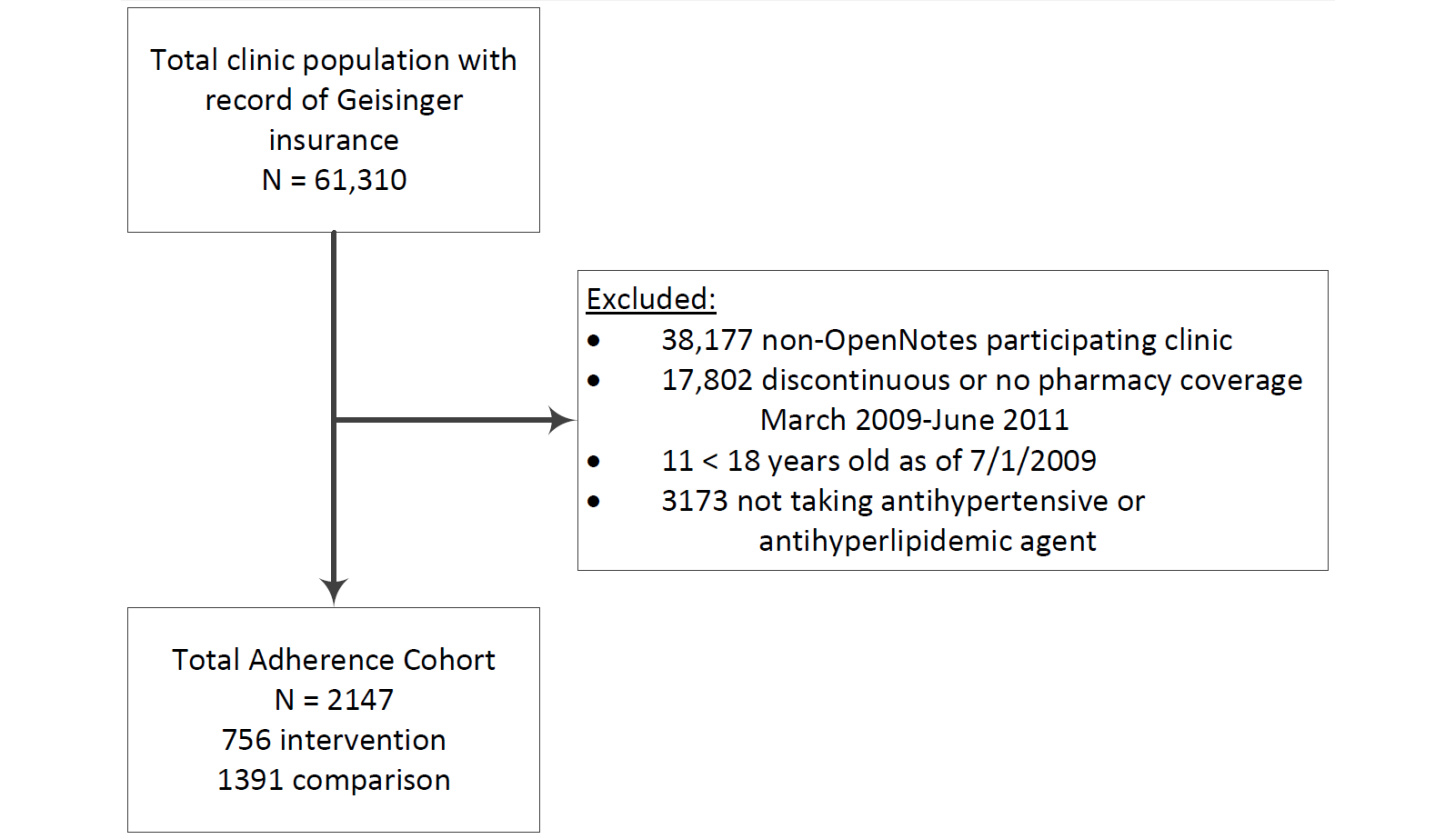
Patient cohort selection.

Patients with GHS insurance cared for by 102 PCPs—24 participating and 78 nonparticipating—were available for comparison. In all, 2147 subjects were eligible and included in our analysis: 756 (35.21%) in the OpenNotes participating group (intervention) and 1391 (64.79%) in the nonparticipating group (control) (see Figure 1).

Characteristics at baseline were similar between the two groups, except for the prevalence of diabetes mellitus and an elevated BMI, both of which were slightly higher in the intervention group (see Table 1). Patients were 59 years of age on average, split about evenly between men and women, and overwhelmingly white.

**Table 1:** Baseline^a^ characteristics for antihypertensive and antihyperlipidemic cohorts.

Variable	Antihypertensive agents			Antihyperlipidemic agents		
	Intervention (n=561)	Control (n=1008)	*P*	Intervention (n=474)	Control (n=913)	*P*
Age in years, mean (SD)	60.5 (12.9)	59.9 (12.7)	.39	60.4 (11.6)	60.6 (11.8)	.74
Sex (female), n (%)	289 (51.5)	554 (54.96)	.19	229 ( 48.3)	466 (51.0)	.33
Race (white), n (%)	556 (99.1)	1001 (99.31)	.67	471 ( 99.4)	908 (99.5)	.84
Hospitalizations in previous year, n (%)	52 (9.3)	89 (8.83)	.77	46 (9.7)	80 (8.8)	.56
Emergency department visits in previous year, n (%)	73 (13.0)	110 (10.91)	.21	61 (12.9)	90 (9.9)	.09
PCP encounters per year, median (IQR)	3.3 (2.3-5.3)	3.3 (2.0-4.7)	.09	3.3 (2.0-5.0)	3.3 (2.0-4.7)	.22
Body mass index (kg/m), median (IQR)	31.6 (28.1-35.9)	31.1 (27.5-35.4)	.08	30.7 (27.3-34.7)	30.1 (26.8-33.8)	.05
Systolic blood pressure (mmHg), mean (SD)	130.8 (12.2)	131.0 (11.5)	.74	127.9 (12.0)	128.4 (11.6)	.43
Diastolic blood pressure (mmHg), mean (SD)	76.4 (8.1)	76.2 (8.0)	.62	75.1 (7.6)	75.1 (7.4)	.91
Total cholesterol (mg/dl), mean (SD)	180.9 (34.8)	184.6 (38.1)	.07	179.8 (38.4)	182.8 (39.6)	.17
High density lipoprotein (mg/dl), mean (SD)	49.6 (14.7)	51.3 (15.1)	.04	48.8 (13.3)	50.3 (13.9)	.06
Low density lipoprotein (mg/dl), mean (SD)	100.8 (29.0)	103.3 (32.1)	.15	99.0 (31.7)	101.6 (32.8)	.16
Hemoglobin A1c (%), mean (SD)	6.8 (1.2)	6.7 (1.0)	.32	6.8 (1.1)	6.8 (1.1)	.54
Charlson Comorbidity Index score, mean (SD)	0.9 (1.1)	0.8 (1.1)	.56	0.9 (1.1)	0.8 (1.1)	.58
Coronary artery disease, n (%)	103 (18.4)	151 (14.98)	.08	94 (19.8)	177 (19.4)	.84
Heart failure, n (%)	36 (6.4)	82 (8.13)	.22	27 (5.7)	63 (6.9)	.39
Hypertension, n (%)	438 (78.1)	794 (78.77)	.75	279 (58.9)	537 (58.8)	.99
Hyperlipidemia, n (%)	321 (57.2)	586 (58.13)	.72	380 (80.2)	749 (82.0)	.40
Diabetes mellitus, n (%)	160 (28.5)	231 (22.92)	.01	137 (28.9)	224 (24.5)	.08

^a^Baseline measurements occurred on March 1, 2010, for pilot providers and July 1, 2010, for remaining participating providers.

^b^Primary care physician (PCP).

^c^Interquartile range (IQR).

^d^Average of up to three most recent readings leading up to intervention start.

^e^Most recent reading leading up to intervention start.

Of the 2147 eligible participants, 818 (38.10%) (281 intervention, 34.4%; 537 control, 65.6%) had a prescription claim for both an antihypertensive and antihyperlipidemic agent, 751 (34.98%) (280 intervention, 37.3%; 471 control, 62.7%) had a prescription claim for only an antihypertensive agent, and 569 (26.50%) (193 intervention, 33.9%; 376 control, 66.1%) had a prescription claim for only an antihyperlipidemic agent in both baseline and follow-up years. Out of 2147 patients, 1569 (73.08%) (561 intervention, 35.76%; 1008 control, 64.24%) were taking at least one antihypertensive agent, and 1387 (64.60%) (474 intervention, 34.17%; 913 control, 65.83%) were taking at least one antihyperlipidemic agent during the baseline and follow-up time periods. Average PDC ranged from 85 to 87% across the 2-year time frame for both antihypertensive and antihyperlipidemic agents (see Multimedia Appendix 1).

Patients in the intervention group were more likely to be adherent to antihypertensive medications compared to the control group (79.7% and 75.3%, respectively)—absolute risk difference, 4.4%; adjusted risk ratio, 1.06 (95% CI 1.00-1.12); *P*=.04 (see Table 2). We found no difference in adherence among those prescribed antihyperlipidemic agents (77.6% for intervention and 77.3% for control)—adjusted risk ratio, 1.01 (95% CI 0.95-1.07); *P*=.86.

**Table 2:** Comparison of patients' adherence^a^ to treatment during baseline and follow-up periods in the intervention and control groups.

Type of agent	Intervention		Control		Unadjusted		Adjusted^b^	
	BL^c^, n (%)	F-U^d^, n (%)	BL, n (%)	F-U, n (%)	BL risk ratio (95% CI), *P*	F-U risk ratio (95% CI), *P*	BL risk ratio (95% CI), *P*	F-U risk ratio (95% CI), *P*
Antihypertensive (n_I_^e^=561, n_C_^f^=1008)	439 (78.3)	447 (79.7)	799 (79.27)	759 (75.30)	0.99 (0.94-1.04), .64	1.06 (1.00-1.12), .04	0.99 (0.94-1.05), .84	1.06 (1.00-1.12), .04
Antihyperlipidemic (n_I_^e^=474, n_C_^f^=913)	372 (78.5)	368 (77.6)	691 (75.7)	706 (77.3)	1.04 (0.98-1.10), .23	1.00 (0.95-1.07), .90	1.04 (0.98-1.10), .21	1.01 (0.95-1.07), .86

^a^Adherent is defined as patients with proportion of days covered (PDC) ≥.80 (percentage with PDC ≥80%).

^b^For antihypertensive agents: adjusted for diabetes, body mass index (BMI), and primary care visits per year; for antihyperlipidemic agents: adjusted for diabetes and primary care visits per year.

^c^Baseline (BL).

^d^Follow-up (F-U).

^e^Number of included patients in the intervention group (n_I_).

^f^Number of included patients in the control group (n_C_).

In a secondary pre/post comparative analysis of antihypertensive users, the percentages of nonadherent to adherent and persistently adherent were 11.2% (63/561) and 68.5% (384/561) in the intervention group, respectively, compared to 9.23% (93/1008) and 66.07% (666/1008) in the control group (see Table 3). Intervention patients had a 27% higher probability of having the outcome of nonadherent to adherent, versus changing from adherent to nonadherent—adjusted risk ratio, 1.27 (95% CI 1.00-1.54); *P*=.048. In a number-needed-to-treat (NNT) analysis, with an absolute difference of 2.0% improvement (11.2% vs 9.2%) from nonadherent to adherent, for every 50 patients offered access to physicians' notes through a Web portal, 1 additional patient will move from nonadherent to adherent to their antihypertensive medication. There was no significant difference between groups in movement from nonadherent to adherent among antihyperlipidemic users—adjusted risk ratio, 0.87 (95% CI 0.65-1.11); *P*=.30.

**Table 3:** Within-person proportional changes and risk estimates in adherence from baseline to follow-up.

Type of agent	Movement from baseline (1^st^ listed) to follow-up (2^nd^ listed)	Intervention, n (%)	Control, n (%)	Unadjusted RR^a^ (95% CI), *P*	Adjusted^b^ RR (95% CI), *P*
**Antihypertensive (n_I_^c^=561, n_C_^d^=1008)**					
	Reference (adherent^e^ to nonadherent^f^)	55 (9.8)	133 (13.19)	N/A^g^	N/A
	Nonadherent to adherent	63 (11.2)	93 (9.23)	1.30 (1.03-1.56), .03	1.27 (1.00-1.54), .048
	Adherent to adherent	384 (68.4)	666 (66.07)	1.05 (1.00-1.09), .05	1.05 (1.00-1.09), .04
	Nonadherent to nonadherent	59 (10.5)	116 (11.51)	1.03 (0.96-1.09), .36	1.04 (0.96-1.09), .30
**Antihyperlipidemic (n_I_^c^=474, n_C_^d^=913)**					
	Reference (adherent to nonadherent)	49 (10.3)	80 (8.8)	N/A	N/A
	Nonadherent to adherent	45 (9.5)	95 (10.4)	0.88 (0.66-1.11), .32	0.88 (0.65-1.11), .30
	Adherent to adherent	323 (68.1)	611 (66.9)	0.98 (0.93-1.02), .44	0.98 (0.92-1.02), .42
	Nonadherent to nonadherent	57 (12.0)	127 (13.9)	0.88 (0.68-1.06), .20	0.88 (0.68-1.06), .20

^a^Risk ratio (RR).

^b^For antihypertensive agents: adjusted for diabetes, body mass index (BMI), and primary care visits per year; for antihyperlipidemic agents: adjusted for diabetes and primary care visits per year.

^c^Number of included patients in the intervention group (n_I_).

^d^Number of included patients in the control group (n_C_).

^e^Adherent is defined as proportion of days covered (PDC) ≥80%.

^f^Nonadherent is defined as PDC <80%.

^g^Not applicable (N/A).

## Discussion

This study is the first large-scale report suggesting that medication adherence to antihypertensive medications improves among patients granted access to review PCP notes through a Web portal. In individual interviews, focus groups, and surveys, patients indicated that being reminded to, and having access to, read their clinicians’ notes lead them to use prescribed medications “better” [1]. This study provides evidence that a cohort of patients invited to review their PCPs’ progress notes through a secure electronic portal demonstrate increased adherence to medications prescribed for hypertension, but not for hyperlipidemia.

Our results are consistent with a smaller randomized study in which 107 heart failure patients received either usual care or access to a secure online medical record that also included clinical notes [4]. Using the general adherence scale, self-reported adherence improved significantly at 12 months in the intervention group. In our study of a larger patient population, information gathered from prescription claims is consistent with that finding when evaluating patients prescribed medications for hypertension.

Why did we find change among patients with respect to antihypertensive therapy, but not with antihyperlipidemic drugs? Prior to undertaking this analysis, in a group discussion with PCPs, including two authors of this study (JD, TD), a group of clinicians hypothesized the findings would more likely demonstrate increased adherence to medications prescribed for antihypertensives than for lipid abnormalities. Reflecting on their own practices, they felt their notes frequently reflected uncertainty about indications for therapy with statins, along with concerns about side effects. In contrast, they felt notes were more definitive about the need for patients to use antihypertensives. Further, pharmacotherapy guidelines for hypertension are widely accepted by clinicians, but there is long-standing and constantly evolving debate about indications for pharmacotherapy following the measurement of lipid levels [17,18]. Do the doctors’ notes convince patients to adhere more closely to antihypertensive regimens, while reinforcing potential ambivalence in both their doctors’ and their own minds when it comes to evaluating and managing lipid levels?

In a post hoc sensitivity analysis of patients prescribed both antihypertensive and antihyperlipidemic agents versus those prescribed either alone, we found a similar magnitude of adherence effect in the antihypertensive and antihyperlipidemic arms regardless of concomitant therapy (internal analysis). In essence, we noted no positive carryover of effect of using antihypertensive agents among those also using antihyperlipidemic agents. This may infer that there is indeed a differential value placed on the benefit of antihypertensive therapy versus antihyperlipidemic therapy. While our findings support our hypothesis, there remains uncertainty as to why a differential effect was found.

Another potential rationale for the difference noted between antihypertensive and antihyperlipidemic groups could relate to the very small decline in adherence from baseline year to follow-up year in either the intervention or the control groups despite evidence that adherence declines over time [19]. We suspect that the inclusion of prevalent antihypertensive and antihyperlipidemic users resulted in this more gradual decline from baseline to follow-up. A drop in adherence to these chronic medications in the first year is well established, but less is known about the adherence changes from one year to the next in populations with long-standing use of such drugs. In a study of elderly patients taking antihypertensive medications, Krousel-Wood et al reported about a 4.3% decline in the rate of adherence per year [20]. We noted a comparable adherence change in the control antihypertensive group, but not in the antihyperlipidemic control arm, suggesting that the antihypertensive control is a more reliable comparison group.

Although we demonstrated that patients who have access to their progress notes have a higher adherence rate to antihypertensive medications, in contrast to the striking patient self-reports, the magnitude of this effect was small. This may reflect limitations in study design deriving from retrospective analysis in a quasi-experimental study not designed or powered to test hypotheses about adherence to medication. We also noted very high adherence levels of our patients throughout the baseline and follow-up periods, perhaps limiting our ability to measure significant changes in adherence. The mechanism by which our patients have improved adherence is largely explained through improved engagement by reading patient notes and/or the reminder to read them. Although this mechanism seems ultimately plausible based upon this evaluation study, it does not specifically target medication nonadherence like other interventions facilitated through patient portals, such as automatic refill requests [21].

Nevertheless, the internal validity of our study is strong, due to the study design and similar comparative groups (see Table 1). We worked to overcome confounding and selection biases by using controls from offices where both participating and nonparticipating physicians cared for patients. In addition, we adjusted our analysis for known and potential confounders and found nothing measurably different from the crude results, suggesting that the comparison group contained characteristics very similar to those known and likely unknown in the intervention group. Finally, we were able to find and measure variables of interest through our electronic and administrative claims database, providing detailed capture of information among our largely nonmigratory patient population.

While the findings are consistent with our initial hypotheses, they may be confounded by factors we cannot measure at baseline. Although not randomized, since the intervention was implemented to patients on the level of the physician, it minimizes, but may not eliminate, unmeasured patient-level differences that could confound results. Differences between groups that developed during the study are more difficult to attribute to confounders unrelated to the interventions instituted as part of rolling out this project. For example, if providers increased contact and interventions with patients more in the intervention arm, this may be due to patients reading their doctors' notes, or it may be unrelated. With OpenNotes being as much a physician-level as a patient-level intervention, this change in practice may impact physician behavior in ways that could influence the outcomes measured in this study, in essence resulting in a Hawthorne effect reinforcing the change in patient behavior we anticipated would occur.

Finally, although not designed or powered to detect differences in clinical end points, medication adherence was identified as a measure of interest during the initial and subsequent phases of this project, limiting bias caused by testing of multiple hypotheses. However, while an appropriate comparator group was identified, there may be baseline differences between the groups beyond those for which we controlled in our adjusted analysis, and this could contribute to the different findings for the two classes of medications. On the other hand, the findings may also underestimate the impact of the intervention, since some patients taking either of the medications in the study group may not have read their PCPs' notes. Overall, 18% of GHS patients cared for by PCPs in the intervention group chose not to read any notes during the approximately year-long study period. While it could be expected that larger effect sizes could be reached by excluding those not having read their notes, we attempted to show the real-world effects of the intervention by including all patients, regardless of note viewing in the final analysis.

Patients reported that reading their clinicians’ notes helps them with their medical regimen, and improved adherence may both improve the quality of care and decrease costs over time, thereby adding value to a system avidly seeking ways to improve care. Albeit carrying modest weight, our findings are consistent with what patients report and what we anticipated. More and wider measurements of these important components of care are urgently required, but for now, the evidence is increasing that fully transparent records can improve communication and engage patients more actively in their own care.

## Appendix

Multimedia Appendix 1 PDC values for antihypertensive and antihyperlipidemic agents.

## Abbreviations

BL: baseline
BMI: body mass index
EHR: electronic health record
F-U: follow-up
GHS: Geisinger Health System
IQR: interquartile range
IRB: Institutional Review Board
N/A: not applicable
nC: number of included patients in the control group
nI: number of included patients in the intervention group
NNT: number needed to treat
PCP: primary care physician
PDC: proportion of days covered
RR: risk ratio
