# Supplementary figures and images for: Sharing Physician Notes Through an Electronic Portal is Associated With Improved Medication Adherence: Quasi-Experimental Study
Source: J Med Internet Res. 2015 Oct 8;17(10):e226. doi: 10.2196/jmir.4872 (PMC4642386; doi:10.2196/jmir.4872)

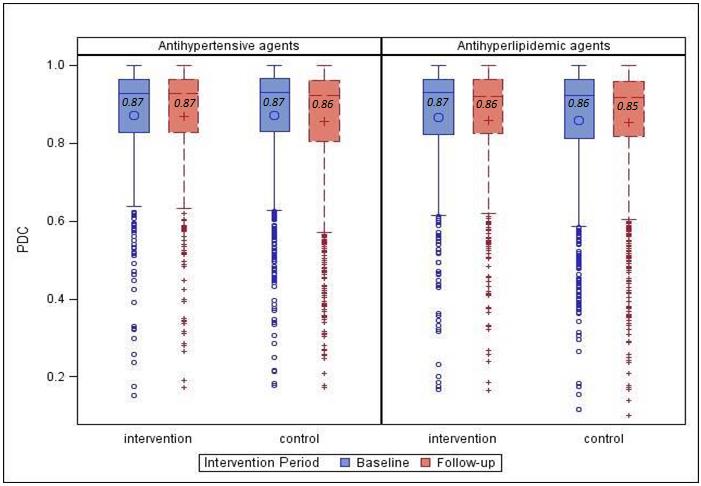

Supplement: Multimedia Appendix 1 [file jmir_v17i10e226_app1.jpg]
